# Menaquinone and Iron Are Essential for Complex Colony Development in *Bacillus subtilis*

**DOI:** 10.1371/journal.pone.0079488

**Published:** 2013-11-04

**Authors:** Gidi Pelchovich, Shira Omer-Bendori, Uri Gophna

**Affiliations:** Department of Molecular Microbiology and Biotechnology, George S. Wise Faculty of Life Sciences, Tel Aviv University, Tel Aviv, Israel; Loyola University Medical Center, United States of America

## Abstract

Cells of undomesticated species of *Bacillus subtilis* frequently form complex colonies during spreading on agar surfaces. Given that menaquinone is involved in another form of coordinated behavior, namely, sporulation, we looked for a possible role for menaquinone in complex colony development (CCD) in the *B. subtilis* *strain* NCIB 3610. Here we show that inhibition of menaquinone biosynthesis in *B. subtilis* indeed abolished its ability to develop complex colonies. Additionally some mutations of *B. subtilis* which confer defective CCD could be suppressed by menaquinone derivatives. Several such mutants mapped to the *dhb* operon encoding the genes responsible for the biosynthesis of the iron siderophore, bacillibactin. Our results demonstrate that both menaquinone and iron are essential for CCD in *B. subtilis.*

## Introduction

In nature, when confronted with difficult environmental conditions, groups of bacterial cells enter a physiological state that conveys new traits to the group and relies on a programmed collaboration between large numbers of cells [1-17]. This collaboration, is known as multicellularity [1-18], and involves sophisticated modes of intercellular communication such as cell-cell physical interactions, long term chemical signals and chemotaxis [1-18]. Examples of such behaviors include fruiting body formation in *Myxococcus xanthus* [6,19-21], and aerial hyphae development in *Streptomyces coelicolor* [22-25]. The Gram-positive bacterium, *Bacillus subtilis*, is also capable of undergoing different multicellular processes including sporulation [7], genetic competence [8], social motility (e.g. swarming) [3,9], extracellular protease production [10,26,27], biofilm formation [11-13,15] and complex colony development (CCD) during spreading on hard agar surfaces [14,16,17]. 

Extensive studies [1,5,14,16,17,28-39] on the properties and dynamics of CCD have been conducted with *B. subtilis* and several species of *Paenibacilli* using a combination of microscopy [28,32,33,35,36], time-lapse cinematography [28,31,37,38] and generic computational modeling [28,31-33,38]. Results from these studies showed that colonial expansion leading to CCD is highly coordinated and cell-density-dependent, involving branches of cells enclosed within a cell-generated polymeric envelope [1,5,28,30,32]. The developing complex colonies were also found to exhibit changes in morphology in response to fluctuations in factors such as media composition, carbon and energy sources and agar concentration [1,28,34]. Computational analysis predicted that CCD in *B. subtilis* and *Paenibacilli* results from the interaction of several forces such as chemotaxis towards an externally supplied food source in combination with two cell-generated forces; a short-range chemo-attractive force between the cells, and a long range chemo-repulsive force [28,31-33,38]. CCD of *B. subtilis* has also been shown to be cell-density-dependent [40]. In addition, mutants blocked in surfactin production were found to be severely impaired in colony expansion and CCD [3,41,42]. The complex spatial structure of surface-associated communities together with gradients of both environmental factors such as nutrients, oxygen and cell generated signals, form different microenvironments throughout the colony [1,15,32,33,36,38,43,44]. These microenvironments ultimately lead to differential gene expression and the development of different cell types in specific areas of the colony [1,15,32,33,36,38,43,44]. 

In prokaryotes, menaquinones (MKs) are low-molecular-weight naphthoquinone derivatives, located in the cytoplasmic membrane, which are involved in electron transfer in respiration [45-48]. All MKs share a 2-methyl-1, 4 naphthoquinone ring, but differ in the structure of the aliphatic side chains attached at the C3-position in the ring structure [45-49]. The side chains of MKs are composed of a variable number of unsaturated isoprenoid residues [45-49]. Hence, MK-related forms are generally designated as menaquinone-*n* (MK-*n*), where *n* specifies the number of isoprenoid units [45,47,48]. The number of isoprenoid residues in the menaquinones side chains can be species-specific, e.g. MK-8 in *Escherichia coli* [50,51], MK-9 in *Corynebacterium diphteriae* [52] and MK-7 in *B. subtilis* [48].

Additional functions of MK, other than respiration, include involvement in the biosynthesis of pyrimidine compounds in *E. coli* [50] and the dehydrogenation reaction of steroid ring A in *Norcardiu restricus* [53]. In *B. subtilis*, MK-7 is also involved in coupled ATP synthesis [54], is required for the glycosylation of certain membrane proteins and is essential for early events in sporulation [48]. 

Microscopy-based observations suggest that some cells, which are closer to the centre of the complex colony, are inactive and sporulate [1,32,33,36,38], similar to what is seen in the formation of fruiting-body-like structures and biofilms by *B. subtilis* [2,12,15,55]. In this regard, we set to investigate whether MK is also required for CCD in the *B. subtilis* strain NCIB 3610 (*B. subtilis* 3610). The results presented in this study point to a role for both MK and iron in multicellular processes such as CCD. 

## Results

### Complex colony development in wild-type *B. subtilis* 3610 strain

**Figure 1:**
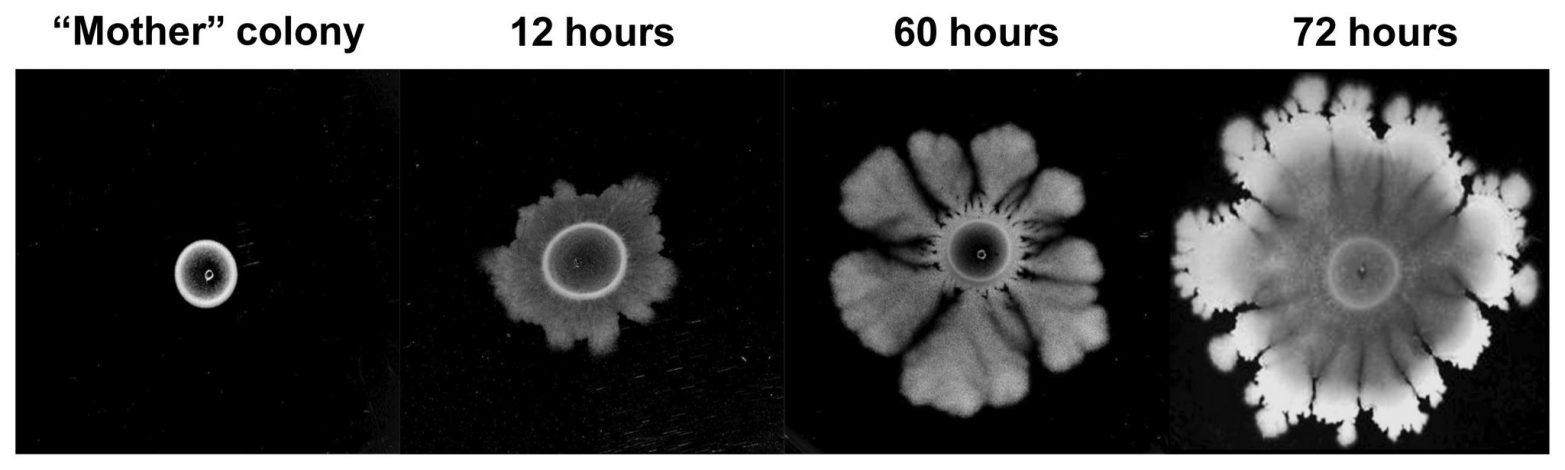
The typical complex colony development phenotype of *B. subtilis* 3610. Approximately 10^5^ cells from the mid logarithmic growth stage were spotted on spreading plates containing 1.2% agar and incubated at 37°C as described in Methods. The plates were photographed at various time points (12, 60 and 72 hours) after inoculation. The presented results are representative of five independent experiments.

When inoculated on spreading plates at 37°C, the cells of *B. subtilis* 3610 first formed a colony at the inoculation site, which we referred to as the “mother” colony (Figure 1). Following 12 hours of growth, branches started to emerge from the edges of this “mother” colony, expanding at rates of 0.3-0.7 mm per hour (Figure 1). Within the first 60 hours of incubation, the branched colony covered most of the plate and 72 hours post inoculation, the cell number increased by four orders of magnitude with a final colony diameter of 4-5 centimeters (Figure 1). The characteristic complex colony consisted of thick branched structures at the periphery reminiscent of irregular dendrite-like structures (Figure 1). The middle of the complex colony contained several layers of cells (15-40 µm thick), while at the outer edge of the plate cells were organized as a monolayer.

### Menaquinone is required for complex colony development in the *B. subtilis* 3610

**Figure 2:**
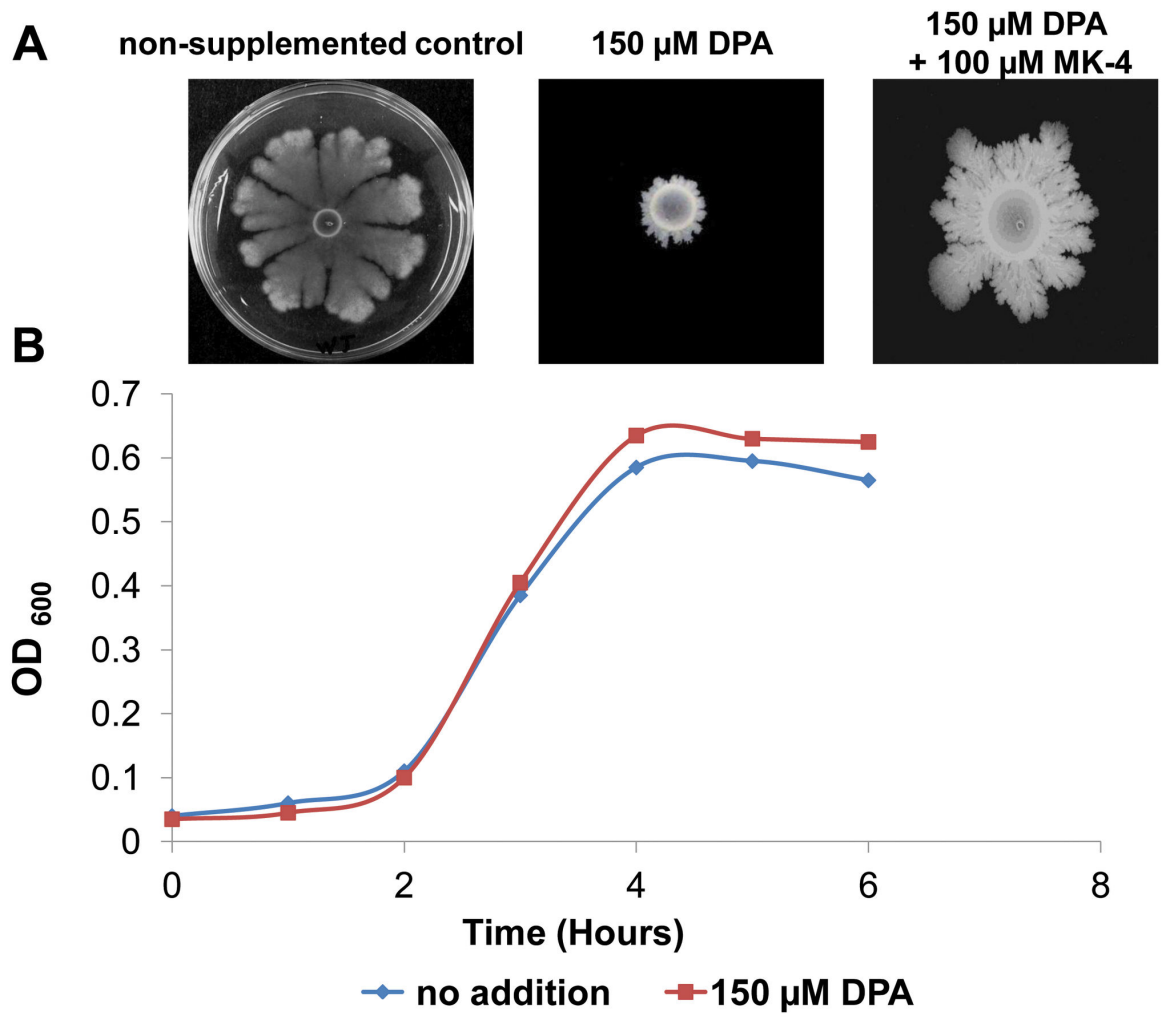
Menaquinone is essential for complex colony development and growth of *Bacillus subtilis*. **A**. Three μl of a fresh overnight culture of the wild-type strain were spotted onto the center of plates containing 1.2% agar supplemented with 150 µM DPA (left) or with 150 µM DPA + 100 µM MK-4 (right). Plates were photographed after 72 hours. Each experiment was carried out in triplicate. **B**. Growth curves of *B. subtilis* 3610 in spreading medium, and in spreading medium supplemented with 150 µM DPA. Each growth experiment was carried out in triplicate.

If MK-7 is indeed necessary for CCD, it is reasonable to assume that blocking its biosynthesis will also inhibit CCD by *B. subtilis*. Blocking of MK-7 biosynthesis can be accomplished by using diphenylamine (DPA), known to inhibit the synthesis of menaquinone in various bacteria [56-58]. In this work, CCD by the parental strain, 3610, was inhibited in the presence of 150 µM of DPA - the diameter of the complex colony was 0.5 cm and did not show irregular dendrite-like structures (Figure 2A). However, this inhibition was reversed when 100 µM of MK-4 was supplemented with the DPA (Figure 2A). Moreover, although CCD was severely inhibited in the presence of 150 µM DPA, growth rate was nearly unaffected (Figure 2B).

Inhibition of MK-7 biosynthesis can also be accomplished by a mutation in a gene directly involved in its synthesis (*men* genes) or in one of the aromatic amino acid synthesis (*aro*) genes [59]. Single colonies of MK biosynthesis*-*defective mutants are smaller compared to wild-type colonies and are also resistant to aminoglycoside antibiotics [54].

To further support the possible role of MK-7 in CCD in *B. subtilis* 3610, we used Mini-Tn*10* transposon mutagenesis in order to isolate mutants defective in MK-7 biosynthesis and examine their ability to develop complex colonies while spreading (Materials and Methods). Eighty-four small colonies (1.75*10^-5^%) were obtained. Forty-two (50%) of them were also resistant to the aminoglycoside antibiotics, kanamycin and paromomycin. Identification of insertion sites of the Mini-Tn10 transposon by sequencing (Materials and Methods) revealed that two (4.76%) of these 42 mutants were defective in the *menG* gene, which encodes a 2-heptaprenyl -1, 4 -naphthoquinone methyltransferase enzyme involved in the final step of the MK-7 biosynthesis of in *B. subtilis* [59,60]. The insertion site, which was identical in both isolates, was located at the beginning of the coding region and therefore the disruption probably resulted in the absence of any functional protein. 

**Figure 3:**
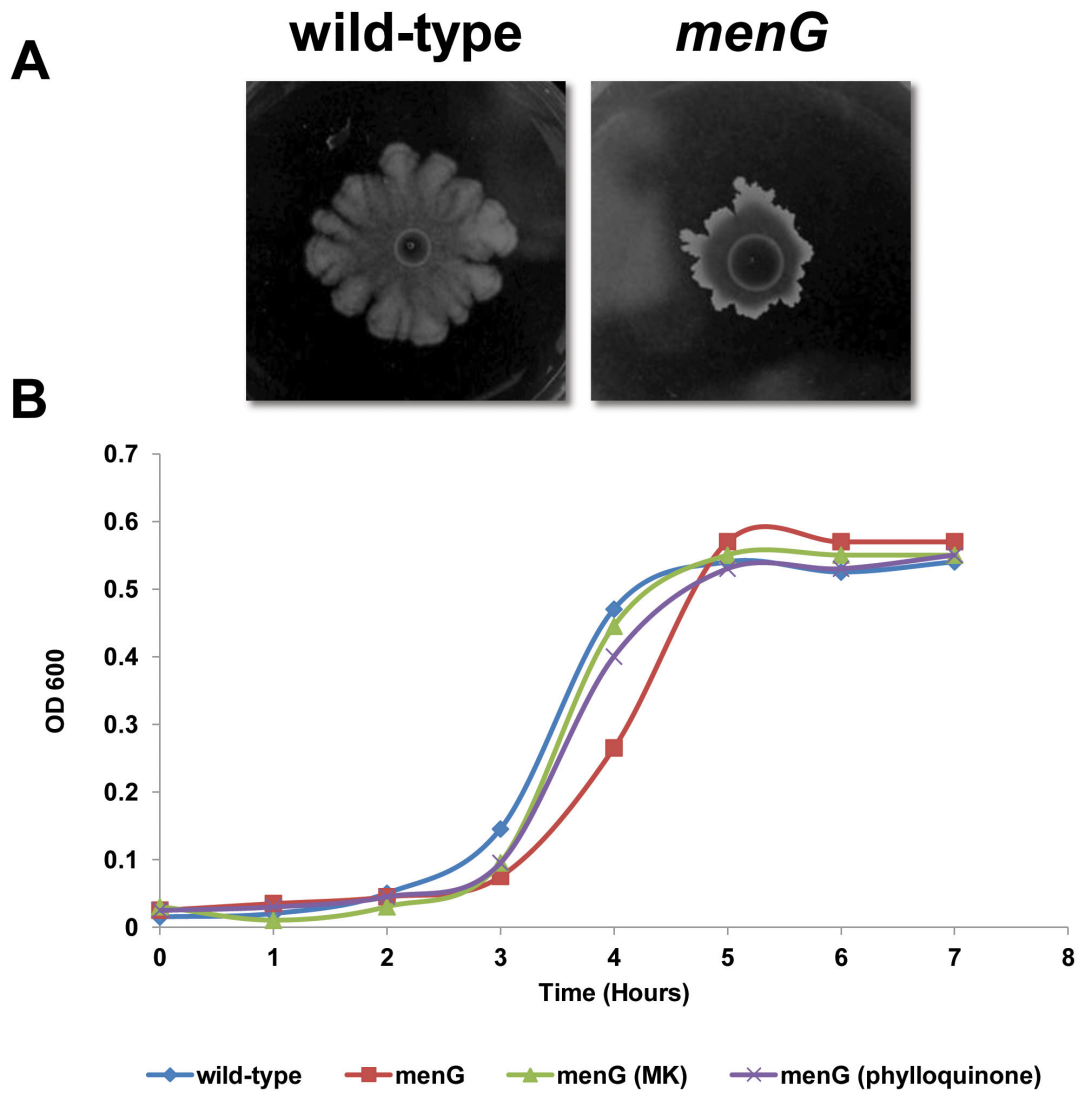
The *menG* mutant is defective in complex colony development and growth. **A**. Cells of wild-type (left) and *menG* (right) strains were spotted on spreading plates containing 1.2% agar and incubated at 37°C in a humid and dark environment. Plates were photographed after 72 hours. The presented results are representative of five independent experiments. **B**. Growth curves were performed in spreading medium or in spreading medium supplemented with 40 μg/ml of either MK-4 or phylloquinone. The presented results are representative of three independent experiments.

As shown in Figure 3A, the *menG* mutants lost their ability to form normal complex colonies. After 72 hours the complex colony reached a diameter of 2 cm and no irregular dendrite-like structures could be observed.

Since CCD is partially driven by cell divisions [42], the effect of a mutation in the *menG* gene on CCD could be explained by growth inhibition. As seen in Figure 3B, the mutation in *menG* caused a substantial growth defect compared to the parental strain. Hence, growth inhibition may explain the inhibition of CCD in this mutant. However, while 40 μg/ml of either MK-4 or phylloquinone did not restore the complex colony forming ability of the *menG* mutant (data not shown), addition of either of these MK-7 derivatives did shorten the generation time from 1 hour to 30 minutes, similar to the wild-type growth rate (Figure 3B). 

### Mutants defective in complex colony development are suppressed by MK-4

**Figure 4:**
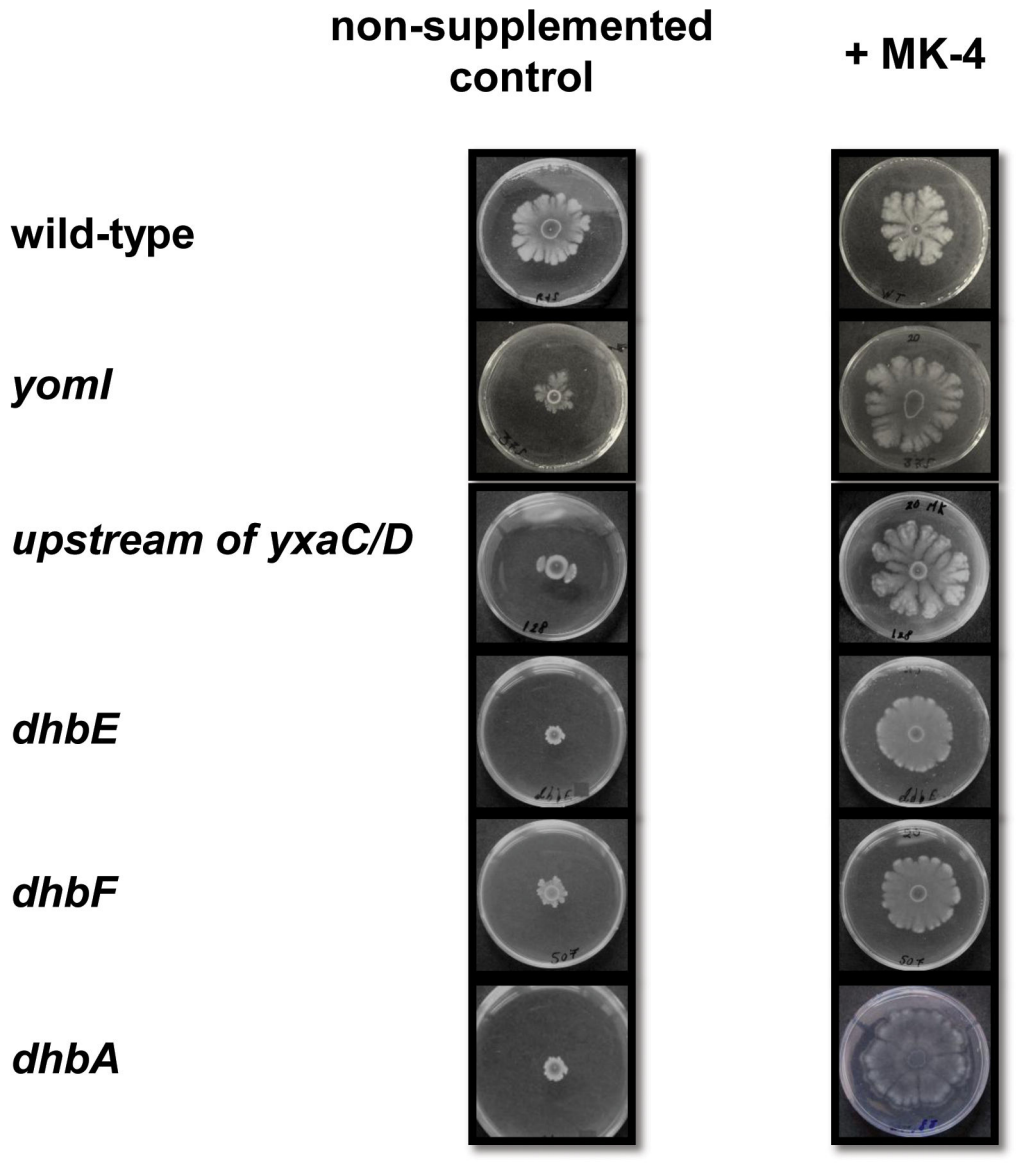
Reconstitution of complex colony development by addition of MK-4. Three μl of a fresh overnight culture were spotted onto the center of spreading plates containing 1.2% agar (left) and spreading plates containing 1.2% agar supplemented with 40 μg/ml MK-4 (right) and incubated at 37°C in a humid and dark environment. Plates were photographed after 72 hours. The presented results are representative of at least five independent experiments.

In order to obtain a larger variety of mutants in menaquinone synthesis, we used an additional random insertional mutagenesis method, which utilizes the Tn*YLB-1* transposon and mutants that can display CCD only in the presence of MK-4 were screened for (Material and Methods). Approximately 17*10^3^ kanamycin resistant colonies from four independent mutagenesis pools were screened for their ability to develop complex colonies on spreading hard agar plates (Material and Methods). Out of 207 mutants that were defective in CCD, 10 mutants (4.8 %) exhibited complex colonies similar to those of the parental strain upon addition of 40 μg/ml of MK-4 (Figure 4). Most of these mutations were mapped to 5 unique genes. Two of these mutants were defective in *yomI*, which encodes a protein similar to a lytic transglycosylase which cleaves bonds in the peptidoglycan [61] and in a non-coding region upstream of a gene cluster consisting of *yxaC*, and *yxaD*, which encode proteins with similarity to peptidoglycan hydrolases, belonging to the LrgB autolysin family, and a probable transcriptional regulator from the MarR (Multiple antibiotic resistance regulators) family, respectively [62]. The eight remaining isolates contained insertions in the *dhbA* (1), *dhbE* (5), and *dhbF* (2) genes, all of which are essential for the biosynthesis of bacillibactin (BB), the known catecholic siderophore of *B. subtilis* that is required for iron-acquisition under iron-limiting conditions [63].

### Mutants in *dhb* genes are defective in bacillibactin synthesis

**Figure 5:**
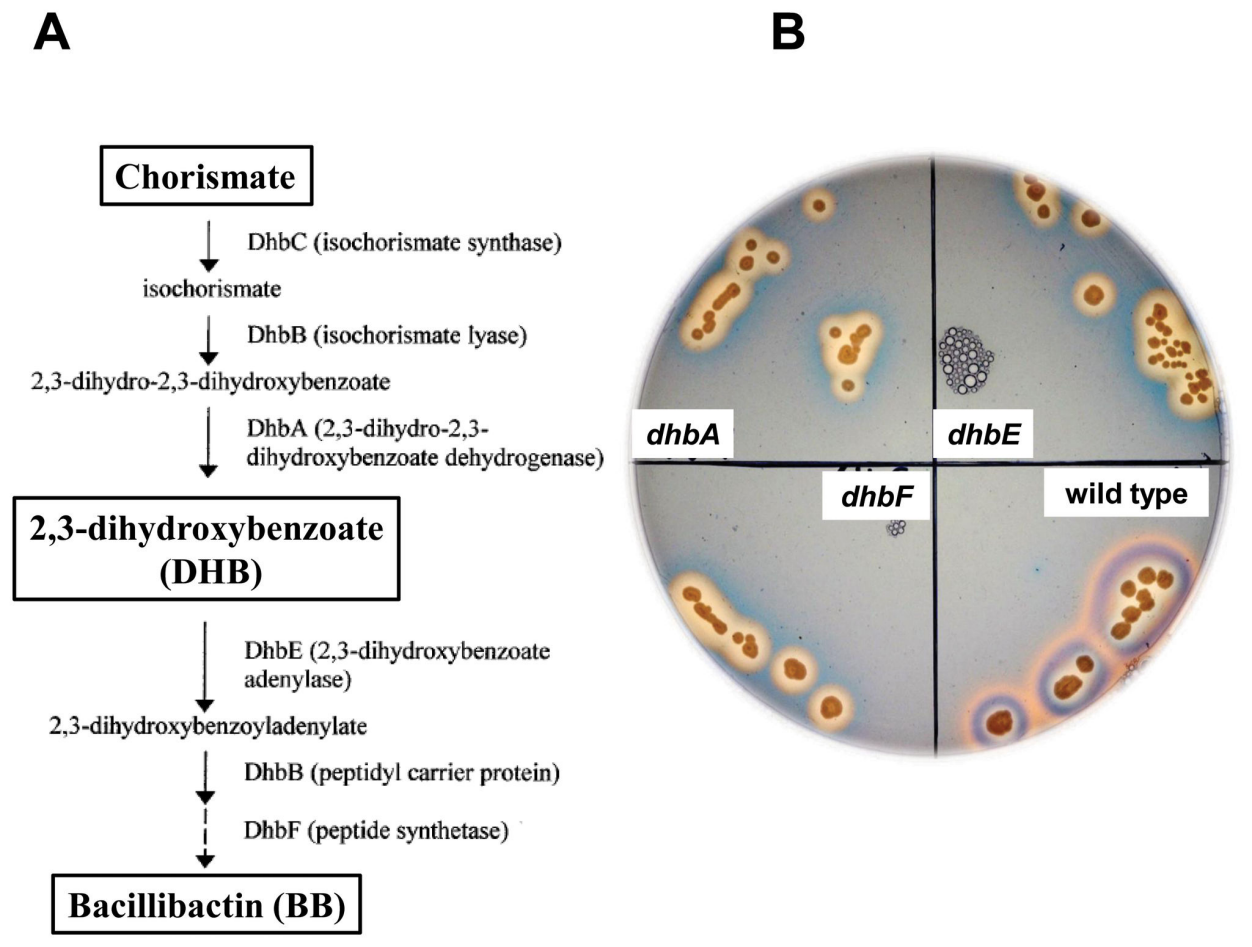
Mutants in *dhb* genes are defective in bacillibactin (BB) synthesis. **A**. Production of BB starts with chorismate and proceeds through the enzymatic activities of the DhbC, DhbB, and DhbA proteins to DHB, an intermediate with weak siderophore activity. DHB is subsequently activated by DhbE-mediated adenylation. A modular peptide synthetase later modifies the resulting 2, 3-dihydroxy-benzoyl-adenylate through the addition of glycine and threonine residues and finally esterifies three of these intermediates to form BB; **B**. Wild-type, *dhbE, dhbF* and *dhbA* strains were plated on CAS hard agar plates (Materials and Methods). Photographs were taken after 48 hours of incubation at room temperature. The results are representative of five independent experiments.

In *B. subtilis* the primary siderophore, BB, is encoded by the *dhb* operon [63-65]. As illustrated in Figure 5A, production of BB starts with chorismate and proceeds through the enzymatic activities of the DhbC, DhbB, and DhbA proteins to the bacillibactin (BB) precursor 2, 3-dihydroxybanzoate (DHB). DHB, an intermediate with weak siderophore activities, is subsequently activated by DhbE-mediated adenylation by forming a 2, 3-dihydroxybenzoyladenylate. In *B. subtilis* 2, 3-dihydroxybenzoyladenylate is then attached to Gly and Thr by the DhbF product to produce the complete and active BB [63-66]. BB is exported out of the cell, and once secreted, binds Fe^+3^ (ferric iron) ions and reenters the cell via an ABC transporter encoded by *feuABC* [67]. Inside the cell the YuiI esterase releases the iron from the siderophore, making it available for cellular processes [67].

In order to confirm that all three *dhb* mutants - *dhbA, dhbE* and *dhbF* - were defective in BB production, a CAS agar assay was performed (Materials and Methods). As shown in Figure 5B, while colonies of the parental strain, 3610, formed dark orange halos as a result of BB secretion and subsequent chelation of the iron present in the medium, colonies of the *dhb* mutants formed significantly paler orange halos. The pale orange halos formed by the *dhb* mutants can be attributed to their ability to produce the precursor DHB, a weaker iron chelator [67]. This result, which is in agreement with a previous report [67], confirmed that all three *dhb* mutants are impaired in the production of BB.

### Reconstitution of complex colony development in the *dhb* mutants by iron supplementation

A significant fraction of the mutations in the CCD-defective strains that were suppressed by MK-4 were localized to the *dhb* operon, which encodes the biosynthesis of the iron siderophore, BB [63-66]. This finding suggests a role for iron in CCD.

**Figure 6:**
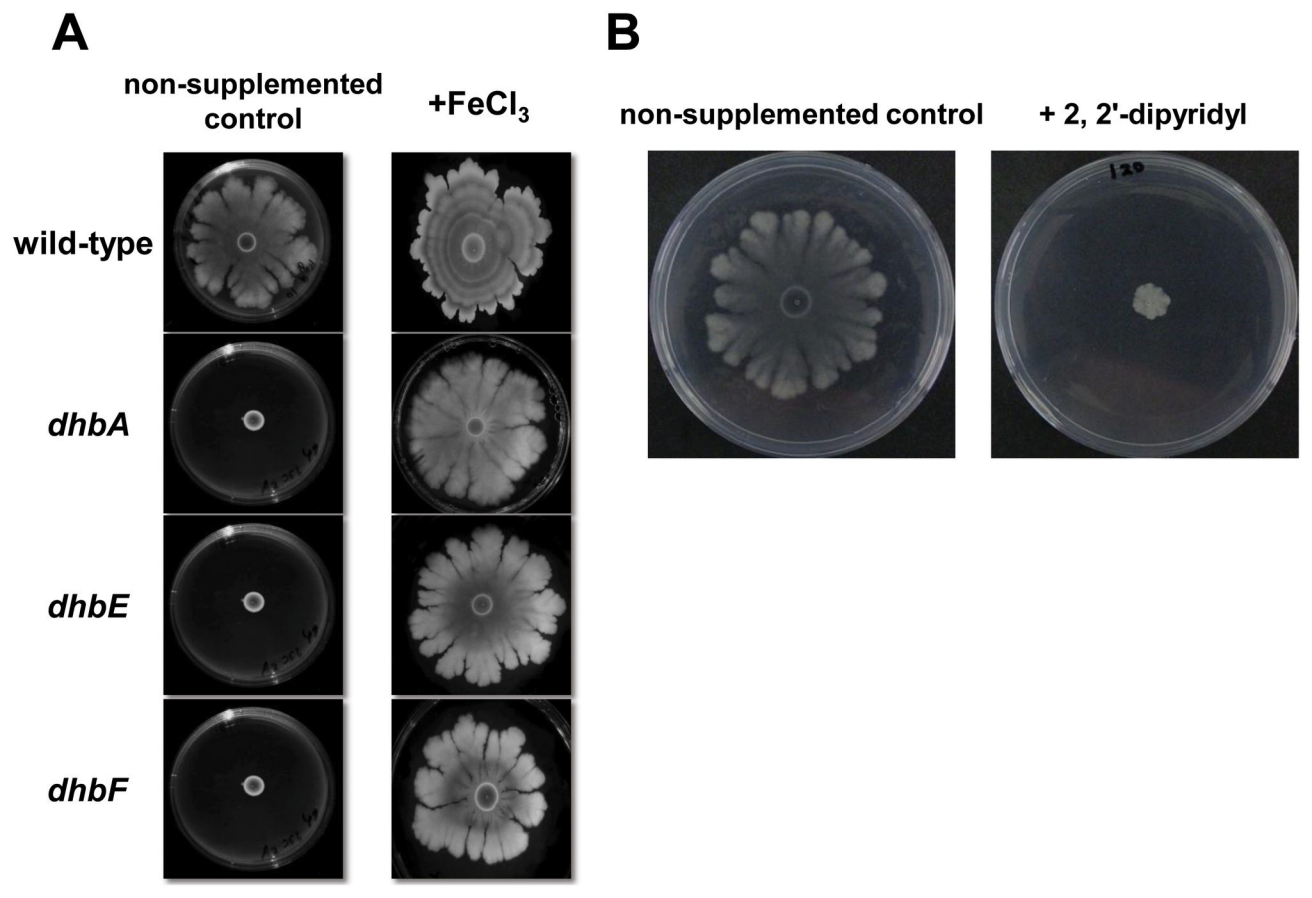
Iron is essential for complex colony development in *Bacillus subtilis*. **A**. Three μl of a fresh overnight grown culture were spotted onto the center of spreading plates containing 1.2% agar (left) and spreading plates containing 1.2% agar supplemented with 150 µM Fe^+3^ (right), and incubated at 37°C in a humid and dark environment. Plates were photographed after 72 hours. **B**. Three μl of a fresh overnight culture were spotted onto the center of spreading plates containing 1.2% agar (left) and spreading plates containing 1.2% agar supplemented with 120 μM of an iron chelator, 2,2'-dipyridyl (right) and incubated at 37°C in a humid and dark environment. Inhibition of complex colony development in the *dhb* mutants in the presence of 120 μM 2, 2'-dipyridyl was observed following 72 hours of incubation at 37°C. The presented results are representative of five independent experiments.

Since the medium used for the spreading plates contained less than 1 µM Fe^+3^, a concentration that is known to stimulate production of BB [63], we were interested in examining the effects of adding Fe^+3^ on the CCD phenotype of the *dhb* mutants. We observed that increasing the Fe^+3^ concentrations in the spreading medium to 150 µM resulted in complete reconstitution of CCD by all three *dhb* mutants (Figure 6A). Furthermore, addition of 120 µM of the iron chelator, 2, 2'-dipyridyl to the medium led to inhibition of CCD in the wild-type strain 3160 (Figure 6B). Taken together these results indicate a role for iron availability in CCD.

**Figure 7:**
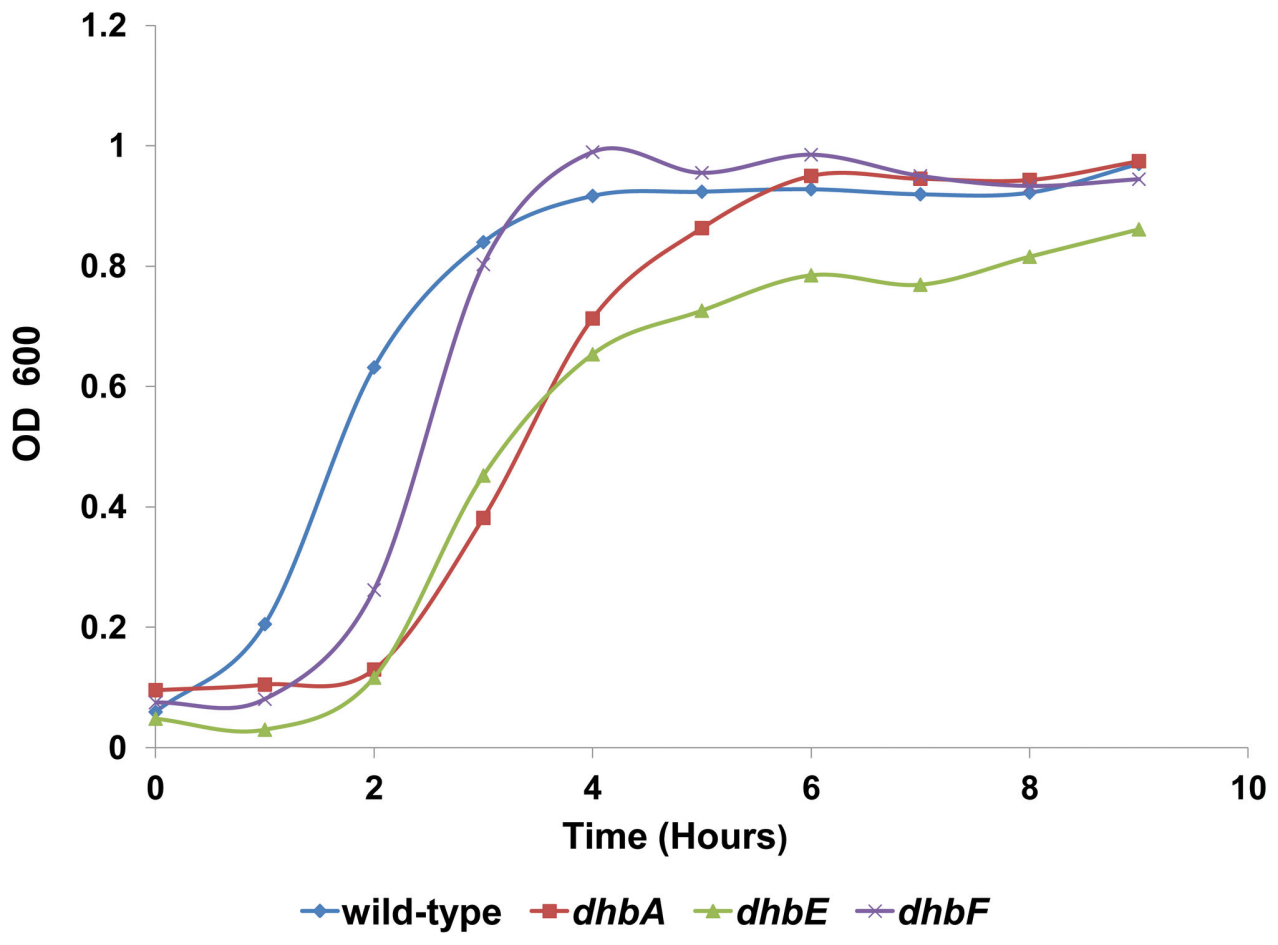
Growth curves of *B. subtilis* 3610 wild-type and *dhb* mutants. Growth curves were performed in spreading medium. The presented results are representative of three independent experiments.

Since iron starvation can prevent bacterial growth [68], we decided to compare the growth rates of the *dhb* mutants with the growth rate of the wild-type, under the condition of iron limitation. When we compared the growth rates (Materials and Methods) of the *dhb* mutants and the wild-type strain, we discovered that all three *dhb* mutations significantly affected growth compared to the parental strain (Figure 7). Calculations have shown that the doubling time of wild-type, *dhbA*, *dhbE* and *dhbF* were 35, 48.8, 49 and 37.5 minutes, respectively. Thus, growth inhibition can explain the *dhb* mutants’ loss of ability to perform CCD.

### The effect of quinone derivatives on the suppression of complex colony development defect in *dhb* mutants

**Figure 8:**
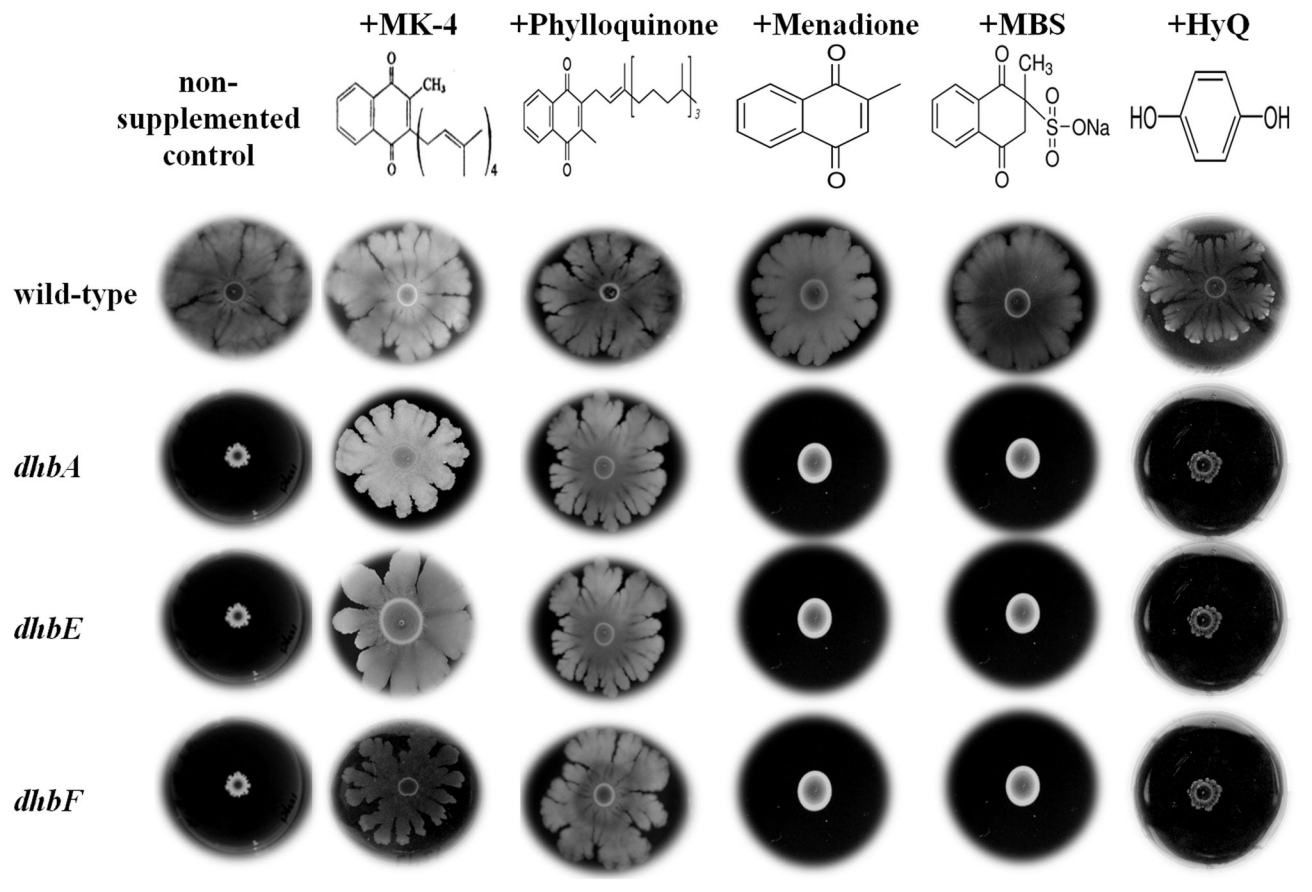
Menaquinone derivatives lacking an isoprenoid side chain cannot suppress the complex colony development defect in *B. subtilis* siderophore mutants. Three μl of a fresh overnight culture were spotted onto the center of spreading plates containing 1.2% agar, spreading plates containing 1.2% agar supplemented with 40 μg/ml of either MK-4, phylloquinone, menadione sodium bisulfite (MBS) or Hydroquinone (HyQ), or spreading plates containing 1.2% agar supplemented with 10 μg/ml of menadione. Photos were taken after 72 hours. Each experiment was carried out in triplicate.

To further investigate the connection between iron availability, MK and CCD, we tested whether other quinone derivatives could reconstitute CCD in the *dhb* mutants. For this purpose, several quinones (Figure 8) were tested for their ability to reconstitute complex colony development in the these mutants (Materials and Methods). Two naphthoquinones, MK-4 and phylloquinone, contain side chains of four and one isoprenoid residues, respectively (Figure 8). Two other naphthoquinones, menadione and menadione bisulfate, lack an isoprenyl side chain as does benzoquinone (HyQ) (Figure 8). As shown in Figure 8, only those quinones that contain at least one isoprenoid side chain restored CCD to the *dhb* mutants while neither of the other naphthoquinones nor the benzoquinone lacking the isoprenoid moiety exhibited any reconstitution of CCD in those mutants.

## Discussion

Complex colony development is a social form of motility that enables bacteria to travel and colonize solid surfaces [1,5,28-39,69]. Despite the extensive description of CCD as a dynamic process in the *Bacillaceae*, little is known of the genes and molecules involved in the organization and conversion of expanding colonies into complex organizations. Since in *B. subtillis* MK is involved in many crucial biological processes, such as respiration [45-49], ATP synthesis [54], and sporulation [70], we set out to investigate whether MK is also required for CCD in *B. subtilis* strain NCIB 3610. For this aim, first, we illustrated that CCD in *B. subtilis* can be inhibited by blocking MK synthesis either by DPA or by mutation in *menG* gene, which is involved in the final step of the MK-7 biosynthesis, and that these mutants resemble other MK-defective mutants studied to date [71-73]. Subsequently, we screened for *B. subtilis* mutants that could form complex colonies only in the presence of MK-4, an MK-7 derivative. In this screen a significant number of independent mutations were mapped to the *dhb* operon, which encodes the genes responsible for BB biosynthesis [74] . Our findings establish a role of MK and iron in CCD by *B. subtilis*. 

CCD motility is considered a passive form of surface translocation that does not require active flagella [42,75]. Being flagella-independent, CCD motility is driven by two forces, operating individually or together. The first is the expansive force of a growing colony caused by cell divisions [42], and the second is a force generated by the swelling of the colony caused by water uptake from the surface [76]. Since spreading motility is partially driven by cell divisions, mutations that affect growth can influence CCD [42]. Since the *menG* and the *dhb* mutants have shown slower growth rates compared to the wild-type strain, the loss of CCD by these mutants can be explained by growth inhibition, which results from insufficient levels of MK or iron. Nevertheless, the finding that the MK-7 analogs, MK-4 and phylloquinone, did restore the growth rate of the *menG* mutant, similar to the wild-type, but not CCD, taken together with our finding that 150 µM of DPA inhibited CCD but not growth, suggest a more specific role for MK in CCD, independent of its role in respiration and growth [45-48,77]. Furthermore, these data imply that higher levels of MK are required for CCD than for growth. In agreement with our observations, previous studies [70,78] showed that a 10-fold higher MK-7 concentration is required for maximum sporulation than was necessary to establish normal cellular respiration, suggesting more specific roles for MK in early sporulation functions. An additional study [47], showed that nearly 50% of the total MK-7 synthesized by *B. subtilis* is secreted into the growth medium at the stationary phase, and thus a large fraction of the MK pool is not involved in respiration. Moreover, since in *B. subtilis*, MK is involved in ATP synthesis [54], it is plausible that as a result of blocking MK biosynthesis, less ATP molecules are synthesized and hence more energy in the form of ATP is required for CCD than for growth. 

Several studies [79,80] have shown that there is coupling of sporulation with multicellular development. For example, in *Myxococcus xanthus*, sporulation apears only after the cells aggregate into macroscopic fruiting bodies [79], and this is also the case for *Streptomyces coelicolor*, which produces spores in raised structures known as aerial mycelia [80]. Moreover, many studies [1,32,33,36,38] suggest that within the complex colony, some cells that are found close to the mother colony can sporulate. Accordingly, one can speculate that sporulation and CCD can be coupled in *B. subtilis*, and since in *B. subtilis* MK-7 is essential for early events in sporulation [70,78], it is possible that MK deficiency inhibits sporolation resulting in inhibition of CCD. 

The findings that in the screen for mutants that perform CCD only in the presence of MK-4, a significant number of independent mutations mapped to the *dhb* operon and that that the iron chelator (2, 2'-dipyridyl) inhibited CCD in the wild-type strain, suggest: 1) a role for iron in CCD in *B. subtilis*; 2) that iron may be essential for MK synthesis in *B. subtilis*; or 3) that there is an interaction between iron and MK. This study is not the first report of iron and siderophores as critical factors in multicellular behaviour. For example it was suggested that a critical level of intracellular iron concentration serves as a signal in *Pseudomonas aeruginosa* biofilm and that an iron uptake system is required for its normal development [68]. Accordingly, we speculate that iron may also serve as a signal molecule that activates iron-dependent pathways that are crucial for CCD in *B. subtilis*. The discovery that defects in CCD in the *dhb* mutants could be suppressed by exogenously added quinones, some of which are not normally used by *B. subtilis*, suggests that iron may be essential for MK synthesis in this bacterium. This assumption is supported by previous studies [78,81-84] that demonstrated that lower iron availability can limit chorismate formation, which is a key step in the biosynthesis of aromatic amino acids as well as bacillibactin and MK-7. Thus, in an environment with low concentrations of ferric ions, the *dhb* mutations can cause an iron limitation that may reduce the levels of the natural menaquinone of *B. subtilis*, MK-7. Thus, the limitation in bioavailability of MK-7 might lead to inhibition of CCD in the *dhb* mutants. Previous works [85-87] showed that menaquinones can reduce ferric iron, supporting the interaction between iron and MK. This could result in a eukaryotic-like vitamin K cycle [88-90], in which the oxidized menaquinone accumulates and cannot be re-reduced because of a defect in the recycling of the reduced MK-7. This MK-7 limitation, which may cause the inhibition of CCD by the *dhb* mutants, could then be overcome by the addition of larger quantities of MK-4 or phylloquinone. The finding that the loss of CCD by the *dhb* mutants could also be suppressed by addition of ferric iron implies that sufficient amounts of iron for normal CCD can be acquired by passive diffusion. 

The molecular specificity observed when testing which exogenous MKs can suppress defects in CCD, suggests a role for the isoprenoid side chain of the naphthoquinone in facilitating CCD, as seen in MK-7. Secretion of MK-7 by *B. subtilis* was suggested to be dependent on the presence of such an isoprenyl side chain [47], and since CCD is known to require other secreted factors such as the protein surfactin [3,41,42], the ability to be secreted may be an important property of MK-7. However, the identity of the protein(s) that require MK as a cofactor and are involved in CCD, and whether they are indeed secreted, will require further study.

## Materials and Methods

### Bacterial strains

All strains used in this work are listed in Table 1. 

**Table 1:** Strains and plasmids used in this study.

**Strains**	**Characteristics**	**Source**
*B. subtilis* NCBI-3610 (3610)	Undomesticated wild-type strain	Bacillus Genetic Stock Center (BGSC)
*menG Mutant of B. subtilis* 3610	*men*G::tn-spc, complex colony development formation defect	This report
*yomI* mutant of 3610	*yomI::*tn-kan	This report
*lysA* mutant of 3610	*lysA::*tn-kan, kan^R^,	This report
*Mutant of* 3610 *carrying an insertion in a non-coding region upstream of a gene cluster consisting of yxaC, and yxaD*	*non-coding region upstream of a gene cluster consisting of yxaC, and yxaD::*tn-kan	This report
*dhb*A mutant of 3610	*dhb*A::tn-kan, kan^R^,	This report
*dhb*E mutant of 3610	*dhb*E::tn-kan, kan^R^.	This report
*dhb*F mutant of 3610	*dhb*F::tn-kan, kan^R^	This report
**Plasmids**	**Characteristics**	**Source**
pIC333	A 2.4-kb mini-Tn*10* transposon containing a ColEl origin of replication, a Spectinomycin resistance gene, a *mls* (Erythromycin + Lincomycin) resistance gene, a thermosensitive origin of replication for Gram-positive hosts (inactive at temperatures higher than 35°C)	[93]
pMarA	A Tn*YLB-1* transposon containing a Kanamycin resistance gene, an Erythromycin resistance gene, a thermosensitive origin of replication (repG+^ts^) for Gram-positive hosts (inactive at temperatures higher than 45°C).	[92]

### Growth Media

Lysogeny broth (LB) medium was composed of 1% tryptone, 0.5% yeast extract, 0.5% NaCl and solidified by addition of 1.5% hard agar. TY growth media for SPP1 phage transduction of *B. subtilis* was composed of 1% tryptone, 0.5% yeast extract, 0.5% NaCl, 10 mM MgSO_4_, and 100 µM MnSO_4_, and solidified by addition of 1.5% hard agar for plates and 0.5% for soft agar. Media for spreading assay (spreading plates) was composed of spreading salt solution (17 g L^-1^ K_2_HPO_4_·3H_2_O, 3 g L^-1^ KH_2_PO_4,_ 2 g L^-1^ (NH_4_)_2_SO_4_, 0.1 g L^-1^ MgSO_4_·7H_2_O), supplemented with 0.5% (w/v) glucose, and 0.025% (w/v) yeast extract. The medium was solidified by addition of 1.2% hard agar. 3ml of the medium were poured onto a 50mm Petri dish and left to dry 3 days at room temperature until plates lost 4-5% of their weight.

### Growth and maintenance

Generally, overnight liquid cultures were prepared by inoculation of a single colony into LB and incubation at 37°C in a New Brunswick gyratory shaker model G-25 at 270 rpm. For long-term storage, overnight cultures were mixed with 25% (^v^/_v_) glycerol and stored at -70°C. Concentration of kanamycin used for supplementing *B. subtilis* growth media was 15μg/ml.

### Isolation of menaquinone-deficient mutants of *Bacillus subtilis* 3610

To generate menaquinone-deficient mutants of *Bacillus subtilis* 3610, Mini-Tn*10* transposon mutagenesis was preformed according to Kearns et al., 2004 [91]. Thirty three single colonies from an overnight streak of *B. subtilis* strain DS1010 (a strain 3610 derivative carrying the temperature sensitive plasmid pIC333 (Table 1)), that were grown on MLS (1 μg/ml erythromycin and 25μg/ml lincomycin) plates were used to inoculate a 1.5ml LB supplemented with MLS. These cultures were rolled at 25°C overnight, diluted 1:100 and transferred to LB supplemented with 100 µg/ml spectinomycin and rolled for approximately 6 hours at 42°C. Candidate menaquinone-deficient mutants of *B. subtilis* were selected by simultaneous resistance to two aminoglycoside antibiotics (11 µg/ml kanamycin and 4 µg/ml paromomycin) when incubated at 37°C. Since, the resistance to aminoglycoside antibiotics stems from the role of menaquinone in the transport of these antibiotics into the bacterial cell [54], bacteria that are resistant to aminoglycoside antibiotics are more likely to be menaquinone-deficient mutants [77]. In addition, defects in electron transport decrease the amount of ATP, leading to a slower growth rate, and consequently MK-defective mutants form small colonies [54]. Accordingly, only small colonies were isolated.

Identification of insertion sites of the mini-Tn10 transposon was performed using the MY051 primer (Table 2) located at the edge of the transposon. 

**Table 2:** Oligonucleotides used in this study.

**Name**	**Sequence (5’→3’)**
IPCR1	GCTTGTAAATTCTATCATAATTG
IPCR2	AGGGAATCATTGAAGGTTGG
IPCR3	GCATTTAATACTAGCGACGCC
MY051	CCCACTTATAAACAAAAGATC
DhbA-Fw	CCTTGGCCTTGAGCTTGCAG
DhbA-Rv	CCTGATTGTTTTGCCTGACG
DhbE- Fw	TTTGAAAAACATCAT
DhbE- Rv	TTCCTCCAGCGTATA
DhbF- Fw	GTGCTGGATGATC
DhbF- Rv	GACGTCCGCCATG

### Random transposon mutagenesis by TnYLB-1

The mutagenesis was preformed according to Le Breton et al., 2006 [92]. A single colony from an overnight streak of *B. subtilis* strain caring the pMarA plasmid grown on 1 μg/ml erythromycin plates was used to inoculate a 3ml LB supplemented with 15 μg/ml kanamycin and 1 μg/ml erythromycin and incubated at 30°C for 14 hours. Subsequently, samples from an overnight culture were plated on spreading plates supplemented with 15 μg/ml kanamycin and incubated overnight at 42°c (restrictive temperature that inhibits plasmid replication). Spreading-defective mutants were picked and isolated. In order to identify the transposon insertion site, genomic DNA from all candidates was purified and digested with *Taq*I. The digested DNA was used for ligation in a concentration that favors self ligation (5 ng/µl). The ligation products were used as a template for PCR using primers IPCR1 and IPCR2 (Table 2). The PCR products were sequenced using IPCR3 primer (Table 2).

### Phage SPP1 generalized transduction

In order to work with isogenic strains it was necessary to transfer the specific mutation to the parental strain *B. subtilis* 3610. Hence, phage SPP1 generalized transduction was preformed according to Kearns et al., 2004 [9]. For lysate preparation, a fresh colony of strain *B. subtilis* 3610 was inoculated in 3 ml TY broth and grown until the culture was circa 0.6 OD_600_. 0.2 ml of cells were mixed with 0.1 ml phage stock suspension and incubated statically at 37°C for 15 min. Three ml of TY soft agar were added and the entire contents were poured onto fresh TY plates. The lysate plates were dried for 20 minutes in a laminar flow hood. Following overnight incubation at 37°C, the top agar was scraped, suspended in 5 ml TY broth, and centrifuged (15 min, 4000 g). The supernatant was treated with 10 µl of 25 µg/ml DNase at room temperature for 10 minutes and filtered. To perform transduction, the recipient colony was inoculated in 2 ml of TY broth and grown until the culture was very dense. One ml of cells were mixed with 10 µl phage stock and incubated statically at 37°C for 30 min. The cultures were centrifuged for 15 min at 4000 g) and the pellets were plated on selective media supplemented with 10 mM sodium citrate. The plates were incubated overnight at 37°C.

### Spreading assay

Three μl of a fresh overnight culture were spotted onto the center of spreading plates containing 1.2% hard agar and incubated at 37°C under conditions of a humid and dark environment. After 72 hours of incubation the plates were examined and photographed.

### Spreading reconstitution of spreading-defective mutants

Spreading reconstitution was monitored using the CCD assay as described above, but the plates were supplemented with 5, 10, 20, or 40 μg/ml of MK-4, and phylloquinone, benzoquinone, menadione or menadione bisulfate. The results shown are for 40 μg/ml of these molecules, except menadione for which 10 μg/ml were used, due to growth inhibition above that concentration. Examination of the reconstitution of CCD in the *dhb* mutants was performed by addition of FeCl_3_ at different concentrations (5, 10, 50, 100 and 150 µM). The results shown are for 150 µM, the only concentration tested that reconstituted spreading fully in all mutants. After 72 hours of incubation the plates were examined and photographed.

### Inhibition of spreading of *B. subtilis* strain 3610 by the iron chelator 2, 2'-dipyridyl

In order to examine the influence of the iron chelator 2, 2'-dipyridyl on spreading, a spreading assay was performed as described above, but the plates were supplemented with 10, 20, 30, 40, 50, 60, 70, 80, 90, 100, 110 or 120 μM of 2, 2'-dipyridyl. The latter concentration was the minimum required for complete inhibition of CCD. After 72 hours of incubation the plates were examined and photographed.

### Inhibition of spreading of *B. subtilis* strain 3610 by diphenylamine

In order to examine the influence of diphenylamine (DPA), which blocks MK synthesis, on CCD, a spreading assay was performed as described above, but the plates were supplemented with 10, 20, 30, 40, 50, 60, 70, 80, 90, 100, 110, 120, 130, 140 or 150 μM of DPA. The latter concentration was the minimum required for complete inhibition of spreading. After 72 hours of incubation the plates were examined and photographed.

### Growth curve analysis experiments

To determine the generation times of all strains, cultures of all tested strains were grown in 3 ml spreading broth at 37°C in 245 rpm. After 10-14 hours, cultures were diluted 1:100 in spreading broth and grown to an OD_600_ of 0.8 at 37°C in 245 rpm. Subsequently, cultures were diluted to an OD_600_ of 0.05 in spreading broth at 37°C in 245 rpm and absorbance at 600 nm (OD_595_) was monitored every 30 minutes. Every growth experiment was performed three times. 

Growth rate experiments were as well performed in spreading broth supplemented with 150 µM of DPA, 40 µg/ml of MK-4, or 40 µg/ml of phylloquinone 

### Chrome azurol sulphonate-hexadecyltrimethylammonium bromide (CAS-HDTMA) 1.5% agar plate assay

An aqueous stock solution of 1.21 mg/ml CAS-HDTMA was prepared, according to the protocol provided by the manufacturer (Sigma). For standard CAS agar plates, CAS-HDTMA solution was diluted 1:10 with standard Davis medium (7 g L^-1^ K_2_HPO_4_, 3 g L^-1^ KH_2_PO_4_, 2 g L^-1^(NH_4_)2SO_4_, 5 g L^-1^ Na_2_Citrate, 1 g L^-1^ MgSO4, 0.2% Glucose) containing 1.5% agar. To test for halo formation of *B. subtilis* 3610 and *dhb* mutants, strains were first grown on LB plates for 10-14 hours at 37 °C. From LB agar plates, single colonies were picked and streaked onto the CAS agar, incubated for 20 hours at 30°C. Further incubation took place at room temperature (between 23-25°C). The plates were scanned once a day to monitor halo formation. Halo intensity was noted after 48 hours.
